# Developing transmission-blocking strategies for malaria control

**DOI:** 10.1371/journal.ppat.1006336

**Published:** 2017-07-06

**Authors:** Robert E. Sinden

**Affiliations:** Department of Life Sciences, Imperial College London, London, United Kingdom; University of Wisconsin Medical School, UNITED STATES

## Background

Analysis of the varied developmental pathways exploited by malarial parasites as they pass through their differing life stages can be challenging [1–3], the parasites often adopting mechanisms that differ from the conventional norms of their eukaryotic hosts. It is, however, these unexpected differences that provide fascination and drive to the basic research scientist and simultaneously expose potential opportunities to intervene and constrain the terrible impact that these formidable adversaries inflict upon their hosts.

The pressures under which current researchers must compete for finite resources may be contributory factors to the disclosure of each fascinating advance in our understanding of *Plasmodium* being reported as if it were the next magic bullet in management of these amazing parasites. This potentially undervalues the beauty of the science done and observations made. One purpose of this article is to review the biology of malaria transmission and to attempt to provide a rational framework for the discovery, design, and application of measures to reduce the prevalence of human malaria infection in endemic communities—here termed “transmission-blocking (T-B) strategies.” Hopefully, it will still highlight the pure excitement of discovery of new biology whilst simultaneously managing expectations as to the utility of the data to contribute to practical control measures. Where possible, lacunae in current understanding that are inhibiting development of effective and sustainable interventions for elimination/eradication of these cunning and resourceful parasites will be identified.

## The purpose and prioritization of T-B strategies

**Fig 1:**
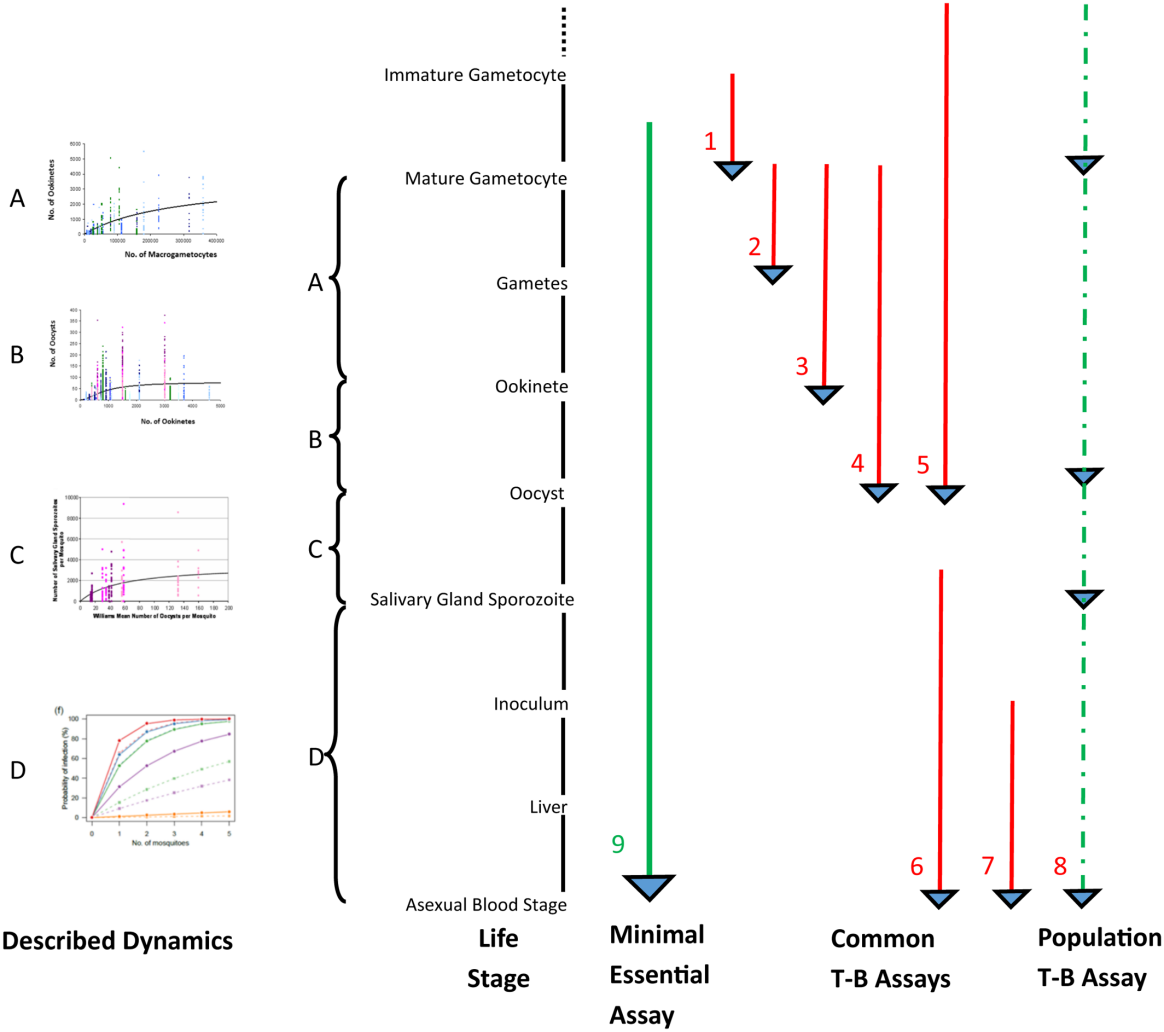
Diagram highlighting the parameters measured by many of the published assays examining the impact of transmission-blocking measures. The biology examined by each assay is indicated by the arrow length; the precise stage enumerated is indicated by the blue arrowheads. The minimal-desired biology to embrace in a directly informative assay is indicated by the solid green arrow; assays measuring less than this ideal are indicated in red. The population assay (dashed green) can be run over repeated cycles of infection measuring multiple desired “endpoints.” The nonlinear dynamic relationships reported between successive transmission life stages are indicated on the left of the diagram. Illustrative references are: A–C [63], D [64], 1 [65, 66], 2 [49, 51], 3–4 [67], 5 [68, 69], 6 [70], 7 [20, 71], 8 [9, 72], 9 [73–75].

The sole purpose of T-B strategies is to reduce the prevalence of malaria infection in affected populations; it is therefore essential to understand how reductions in each developmental phase of transmission relate to this key endpoint (See Fig 1). It is reassuring that Griffin, using current modelling techniques, recently computed that there is no bistable equilibrium in transmission and therefore that elimination is feasible [4]. The following discussion is, however, founded upon one of the earliest models of malaria transmission, the Ross-MacDonald relationship [5–7], which clearly identifies the impact of key component events regulating malaria case prevalence over time, i.e., the basic reproductive value, R_o_.

$$\text{Ross}/\text{MacDonald formula}\ldots \ldots \ldots \ldots \ldots .Ro=\frac{ma^{2}bp^{n}}{-rlog_{e}p}$$

The component terms can simply be ranked by their descending potential “impact” upon R_o_ from the highest, p, the daily survival of the infected mosquito (p^n^), where n can be >9, through a, the human biting rate (a^2^), to the lowest trio: m, the mosquito number relative to man; b, the probability the infected mosquito will infect a human host; and r, the survival of the infectious human host. Thus, as parasitologists, we must appreciate that reducing either the survival of the infected female anopheline or the mosquito biting rate will have greater impact than reducing either the infectivity to “r” or from “b,” the mosquito. Interestingly, this 60-year-old prediction was largely endorsed in a recent analysis of the impact of insecticides (long-lasting impregnated bednets, indoor residual spraying [LLIN, IRS]) and antimalarials (artemisinin combination therapy [ACT]) [8]. Despite the predicted low-ranking impact of targeting “r,” it is encouraging that an intervention described as being only partially effective, i.e., the antimalarial drug (atovaquone), which reduced gametocyte-to-oocyst conversion by only 57%, can eliminate infection in a low-transmission setting (<3 bites/cycle) but notably has no measurable impact in a high-transmission setting (above 4 bites/cycle) [9].

A common feature of parasitic infections is the sheer number of organisms involved, numbers that often overwhelm a host’s ability to control; the asexual blood-stage infections in malaria are 1 example. These vast populations, e.g., 10^10^–10^11^ individuals in a single host, have the potential to display such diversity in phenotype that they “outwit” either the human immune response by antigenic variation [10] or antigenic diversity [11], or the pharmaceutical industry by random mutation. When considering malaria transmission, the numbers of parasites (and therefore the variants/mutants challenged) can be reduced by around 7 orders of magnitude (10^3^ macrogametocytes/mosquito or 10^3^ sporozoites/bite versus 10^10−11^ asexual parasites/human host). Thus, one of the major long-term advantages in developing T-B strategies will lie in the comparatively small populations of parasites actually challenged by the intervention. This fact alone must considerably increase the impact or extend the useful life span of any intervention targeting these bottleneck populations (gametocytes and liver schizonts).

Noting the variation of parasite number “within” the life cycle, it is unclear why we do not make more use of the variations in host or vector numbers to improve the efficiency of our efforts expended on parasite control. Transmission is often seasonal, a pattern imposed by the cyclic changes in the populations of mosquito vectors. Whilst it is unclear whether the parasite is transmitted at all in the “dry” season, overall transmission requires the long-term survival of either or both infected vectors and hosts. Interesting data has recently been presented suggesting that the increased frequency of mosquito feeding itself enhanced the infectivity of “overwintering” parasites by promoting gametocytogenesis [12]. Should we consider attacking the vector (both their abundance and their vectorial capacity), i.e., the potentially infectious reservoir, during the “dry/low” season [13, 14]?

## The design and application of T-B measures directly targeting the parasite

The relevant parasite biology extends from the mature gametocyte circulating in peripheral bloodstream until the invasion of the hepatocyte in the subsequent host. With current technologies, vaccines targeting the extracellular parasites (egressed gametocyte, gamete, ookinete) prior to invasion of the mosquito midgut wall [15–18] and the sporozoite in the recipient host [19, 20] are the focus of attention. Whilst the limited duration and partial efficacy of the current pioneering antisporozoite vaccine (RTSs, which target the circum-sporozoite protein [CSP]) are both frustratingly low [21–23], we should not forget that potential measures that may be partially effective in a single cycle of transmission can, if successfully applied over many cycles of transmission, have significant impact [9]. Thus, the duration of an effective response may prove to be a more useful property than absolute efficacy for a T-B vaccine. Progress on vaccines targeting infection of the mosquito has in recent years moved forward apace, vaccines targeting gametocyte/gamete surface proteins (e.g., pfs230, pfs48/45, HAP2) [9, 16, 17, 24–27] and, notably, pfs25 on the macrogamete and ookinete are already displaying useful efficacy levels [28], and pfs25 has been produced in a wide range of expression systems with diverse utility [29–31]. Noting the practicality of delivery of subunit vaccines (as opposed to live parasites, e.g., [32, 33]) in mass-administration campaigns, if the duration of response can be improved, theoretical and laboratory experiments suggest these vaccines have the most useful contribution to make in elimination campaigns, particularly in combination, e.g., pfs25 and CSP [34, 35].

By contrast, with the slow development of T-B vaccines, the recent focus on the discovery and development of T-B drugs targeting the processes of infection of the mosquito has been remarkable, and the recent decision of MMV to formalise the development of compounds specifically targeting transmission (TCP-5) [36] is to be welcomed. Since “promotion” of the concept [37], the development of assays to identify compounds inhibiting parasite sexual and sporogonic development has flowered [38–53]. Current analyses reinforce conclusions reached some 30 years previously [54–56] as to the limited cellular strategies of some transmission stages. The metabolism of the immature sexual stages (male and female) is largely similar to the asexual parasites [57, 58], whilst the mature gametocytes are metabolically down-regulated (insensitive to many classes of schizonticide) [58], and the mature male and female differ markedly in their drug sensitivity. The female is sensitive to approximately a quarter the number of compounds inactivating the male [49]. By contrast, gametogenesis and microgametogenesis in particular offer numerous potential novel targets for intervention, and the ookinete exhibits a wide range of molecular pathways not seen in the blood-stage parasites (personal communication, M.J. Delves to MJD-RES research group). This knowledge will lead to the rapid identification of new targets.

A person infected with *Plasmodium falciparum* will be prompted to seek treatment at the time of fevers (due to the rising asexual blood-stage infection), but the infectious gametocytes will usually peak 8–10 days later [59]. By contrast, in *P*.*vivax* and the other 3 species infecting man, these 2 populations are contemporary. Consequently, it is only in the case of *P*. *falciparum* where the selection of T-B compounds of practical merit will be compromised by the problems of their effective delivery to the slow-maturing gametocyte population. Delivering drugs to kill the parasite in the blood meal or later in the sporogonic period of development (target candidate profile 6 [TCP6] of the MMV portfolio) is even more problematic; neither the time between dispensing the drug to the patient and its uptake by the insect nor the amount ingested by the vector can be controlled, making pharmacokinetics/pharmacodynamics (Pk/Pd) optimisation difficult. So, how does one “package” the effective delivery of drugs to this “late” gametocyte population? One remedy is straightforward: deliver both the schizonticide and a gametocide (with either a very long half-life or, preferably, irreversible activity) simultaneously—the schizonticide will kill all asexuals and young gametocytes up to day 6 of development, and the gametocytocide (here defined as a drug rendering all stage V gametocytes noninfectious to the vector) will sterilize all older gametocytes, which at the time will either be in the peripheral circulation or in the bone marrow [60–62].

## The missing pieces in our jigsaw of knowledge and tools

### Drugs

Here we are found “between a rock and a hard place!” The most accessible target (the mature gametocyte) is metabolically 1 of the 2 least active stages of the life cycle. We have 2 solutions: (1) we find methods to improve the “longevity” of the T-B drugs such that we can reliably attack the reactivated and highly vulnerable gametocytes when they are undergoing gamete formation and then ookinete formation in the mosquito midgut (a benchmark objective might be to develop a drug active for at least 10 days following delivery to the sick patient) and (b) we analyse in depth the limited targets that may be inhibited irreversibly in the repressed mature stage V gametocytes (only in the past 3 years have modern technologies been applied to this objective [57, 58]). Current functional assays have revealed numerous inhibitory compounds with which to probe the latter question.

The only viable method to inhibit sporozoite development with drugs is to prevent migration from the infected bite to the liver. Whilst automated screens for such drugs exist [76], it must be asked whether their delivery to entire populations at risk is viable.

### Vaccines

Vaccines targeting sporozoite transmission are the most advanced currently available, whether as subunit vaccines RTSs [77] or whole/live attenuated parasites [20, 33]. We remain, however, remarkably ignorant of the molecular foundations of sporozoite infection.

On the massive generalization that T-B vaccines act by antibody interference of the biological function of accessible parasite surface molecules in the mosquito midgut, we must candidly admit that we do not yet understand (1) the highly vulnerable biology of fertilization (gamete recognition and fusion), (2) parasite defences against natural attack by host and vector immune factors, nor (3) the mechanisms by which the ookinete recognises and invades the midgut epithelium. Whilst efforts to address these lacunae in our knowledge have been made [78–82], these remain verdant pastures for future study. Whilst the limited spectrum of available vaccine candidates is being pursued successfully, to develop vaccines of high utility in population-wide delivery programmes, we need to improve vaccine immunogenicity and, perhaps more importantly, the longevity of the effective immune responses raised, whatever target we choose.

### Novel interventions

Whilst drug and vaccine development provides myriad opportunities for intervention, we need to be alert to novel and disruptive intervention concepts. Currently, such ideas are all based on indirect methodologies acting through the vectors and therefore lie outside the remit of this brief article. They include transgenic approaches to reduce mosquito biting rates (1) by disrupting the vector host-seeking behaviour [83, 84], (2) by up-regulating mosquito factors that suppress parasite infectivity (such as oxidative responses [85–89]), and (3) by impacting mosquito reproduction [13, 14]. Have we asked whether we could similarly seed selectively induced lethal/semilethal phenotypic characters into parasite populations, or does the clonal complexity of parasite population structure preclude this approach [90]? One novel approach has recently been proposed—that of perturbing the pattern of gametocyte sequestration and release into the peripheral bloodstream by (inter alia) modulating the deformability of the maturing gametocytes [91], an approach that will only be feasible when we overcome the problem of sustained-intervention delivery.

### Understanding the measurement of impact

If we are ever going to apply T-B measures efficiently, we must understand their performances in the field with respect to the key deliverable in any elimination campaign, namely, the reduction in infection prevalence in the vertebrate host. Those working on the biology of malaria transmission have in the past conducted a wide range of assays to determine the efficacy of parasite transmission to and from the mosquito; these assays have diverse endpoints and often-unappreciated variability (Fig 1). Outputs measured have included gametocyte abundance through ookinete, oocyst, and sporozoite production in the vector, density of exoerythrocytic schizonts in vitro, and, more rarely, blood-stage parasitaemia and prevalence in vivo. Most of the assays, whilst invaluable in the development of our understanding of the biology of transmission, fail to provide the key readout (reduction in infection prevalence) for rational decision making. The fundamental basis for this critique is that the more biology that intervenes between assay readout and the desired endpoint (here, infection prevalence in the vertebrate population), the more complex the relationship with the parameter measured (see Fig 1). For example, recall the 60-year discussion on the relationship between the limited steps linking gametocyte number/patency and the infectious reservoir [75, 92–100] or between salivary gland sporozoite number and infectious potential [64, 101, 102]. Indeed, a widely used parameter of vector infectious potential, the entomological inoculation rate (EIR), fails to reflect mosquito sporozoite burden at all. Often, it is argued that readily accessible outputs are the best/most cost effective to measure—best, rarely; cost effective, not if they lead to erroneous generalizations! The only current assay for T-B interventions that embraces the entire biology of transmission and provides the desired output is the population transmission assay [9]. Despite measuring the key parameters of parasite infection prevalence over many cycles of transmission, its dependence upon the rodent parasite *P*. *berghei* does mean the results will need to be fine-tuned for each of the 5 species infecting man [103]. However, we should anticipate that it will provide an understanding of the core qualitative relationships between sequential transmission life stages—relationships that may vary quantitatively between the different parasite species. Importantly, the assay will permit the direct comparison of diverse interventions targeting different life stages of the biology of transmission. With these core relationships identified, it will be possible to ask focussed, rationally prioritised questions in human populations in endemic areas, questions that are orders of magnitude more expensive to address. Amongst the lessons already learnt from the population assay [9] are (1) the saturated nature of malaria transmission means that the magnitude of impact of any intervention declines with increasing transmission intensity. Thus, in human studies, the transmission intensity of the test site must not prejudice the ability to measure meaningfully the impact of the intervention under test; it is possible this has happened in the field evaluation of some antimalarial drugs, (2) even partially effective measures can, over multiple cycles of transmission, eliminate the parasite from the population in low-transmission settings, and (3) the higher the transmission intensity, the higher the efficacy of the intervention required to achieve measurable reductions in transmission in finite periods, e.g., the single cycle of transmission often used.

### Combining T-B measures

Recognising that even the limited repertoire of T-B measures attacks different parasite properties by diverse mechanisms, it is logical to ask whether their combined use is additive, competitive, or synergistic. An obvious combination that can be studied today is that of a combined T-B and sporozoite vaccine (Pxs25/CSP) [34, 104] to ask whether there is advantage in hitting both bottleneck populations in the same cycle of transmission. Equally, combinations of drugs or vaccine/drug combinations can be modelled and should be considered.

## Concluding remarks

*Plasmodium* has evolved to be one of the most successful parasites of man. Consequently, it offers one of the most challenging adversaries for the enquiring scientific mind. Success in unravelling these hidden strategies is reward in itself, both for the fortunate investigator and the research community at large. Any aspirational young scientist should be encouraged to share this challenge. However, just a fraction of the discoveries made will lead to the design of successful interventions. To use our limited resources effectively, it is incumbent upon both researchers and developers to identify and, based on secure understanding of the biology of the parasite, dispassionately prioritise those lead concepts. Hopefully, this brief overview may help in setting the agenda for the integration, interpretation, and application of our collective understanding of malaria transmission and to develop what have the promise to be powerful tools in the elimination and perhaps eradication agenda.
